# Trade-Offs and Synergies Among Ecosystem Services Influenced by Forest Type and Their Implications for Spatial Management in the Upper Minjiang River Basin, China

**DOI:** 10.3390/plants15142149

**Published:** 2026-07-12

**Authors:** Lifang Hong, Guochun Zhang, Nan Cong, Mengyuan Bai, Ping Ren, Jiangtao Xiao

**Affiliations:** 1 Key Lab of Land Resources Evaluation and Monitoring in Southwest China (Ministry of Education), School of Geography and Resource Sciences, Sichuan Normal University, Chengdu 610066, China; honglifang@stu.sicnu.edu.cn (L.H.); baimengyuan@stu.sicnu.edu.cn (M.B.); renping@sicnu.edu.cn (P.R.); 2 Sichuan Provincial Institute of Forestry and Grassland Inventory and Planning, Chengdu 610081, China; zgc_430@163.com; 3 CAS Key Laboratory of Ecosystem Network Observation and Modeling, Lhasa Plateau Ecosystem Research Station, Institute of Geographic Sciences and Natural Resources Research, Chinese Academy of Sciences, Beijing 100101, China

**Keywords:** forest ESs, trade-offs and synergies, spatially constrained K-means, Upper Minjiang River Basin

## Abstract

The Upper Minjiang River Basin is a critical ecological barrier in the upper Yangtze River, where forest ecosystems play a vital role in carbon sequestration, water conservation, and soil retention. Given that different forest types exhibit significant variations in community structure, species composition, and ecological processes, their ecosystem service (ES) supplies and trade-off/synergy relationships are also expected to show distinct heterogeneity. However, systematic research on the trade-offs and synergies of ESs across different forest types remains limited, constraining the development of precision forest management and differentiated management strategies. To deal with this, we used the InVEST model and calculated five key services across the basin: carbon stock (CS), water yield (WY), soil conservation (SC), habitat quality (HQ), and forest stock volume (FSV). We then applied Spearman’s correlation, root mean square deviation (RMSD), and the GeoDetector model to analyze trade-offs and uncover driving mechanisms. Finally, we used spatially constrained K-means clustering to map different management zones. The results indicate that the Upper Minjiang River Basin stored 1.78 × 10^8^ t of carbon, retained 2.98 × 10^8^ t of soil, produced 6.48 × 10^9^ m^3^ of water yield, maintained a mean habitat quality of 0.78, and supported a forest stock volume of 1.20 × 10^8^ m^3^. Coniferous forests exhibited the highest CS (181.07 t ha^−1^) and FSV (176.37 m^3^ ha^−1^), whereas shrublands contributed the largest share (52.17%) of regional water yield. At the regional scale, CS and FSV showed the strongest synergy (r = 0.71, *p* < 0.01), while WY displayed significant trade-offs with most other services. GeoDetector analysis revealed that forest type acts as the primary driver shaping the relationships among services, while elevation and precipitation play supporting roles. Based on the ES bundles identified via spatially constrained K-means clustering, the Upper Minjiang River Basin was divided into four distinct management zones: a carbon sequestration core zone, an ecological balance zone, an ecologically fragile zone, and a multifunctional conservation zone. Therefore, findings from the Upper Minjiang River Basin may provide insights applicable to other mountain forest ecosystems facing similar environmental and management challenges.

## 1. Introduction

The provision of ecosystem services (ESs) and ecological sustainability are being increasingly reshaped by global climate change and human activities [1,2,3]. The Millennium Ecosystem Assessment (MA) reported that human demand for ESs has exceeded nature’s carrying capacity, resulting in widespread ecosystem degradation, particularly in regulating and cultural services [4]. These services are closely interconnected because they depend on shared ecological processes and limited natural resources. Consequently, enhancing one service may reduce the supply of another (trade-off), whereas multiple services may also increase simultaneously (synergy). Understanding these interactions is essential for balancing ecological protection with socioeconomic development and has become a central theme in ecological research [5,6,7,8,9]. Among terrestrial ecosystems, forests are particularly important because their complex vegetation composition and stand structure strongly regulate key ecological processes such as carbon accumulation, hydrological regulation, and soil stabilization [10,11], thereby influencing the magnitude and direction of ES trade-offs and synergies.

These ecological processes are particularly pronounced in subalpine forests, where coniferous forests (CF), shrublands (SL), and broad-leaved forests (BF) are distributed in a mosaic pattern along elevation gradients. Consequently, ES relationships exhibit stronger spatial heterogeneity and scale dependence. Early studies primarily treated forests as a land-use type, focusing mainly on change processes and landscape pattern responses [12,13,14]. Subsequent research gradually shifted toward the quantitative assessment of ESs, employing model-based approaches to conduct spatial analyses of single or multiple service categories. For example, in some regional studies, coniferous and broad-leaved mixed forests (CBMF) typically demonstrated synergistic relationships among water conservation, SC, and CS, whereas areas dominated by either pure CF or BF were predominantly characterized by trade-offs among these services [15]. A study conducted in the Funiu Mountain region found that, at the forest-type scale, except for *Pinus massoniana* forests, the four ESs in other forest types exhibited synergistic relationships to varying degrees [16]. Therefore, this study takes forest type as the fundamental analytical unit to reveal how forest-type heterogeneity regulates ES interactions. This perspective provides a more direct scientific basis for precision forest management and differentiated ecological restoration.

The Upper Minjiang River Basin, located in the mountainous region of southwestern China, serves as a critical ecological barrier in the upper reaches of the Yangtze River and constitutes a core segment of the regional ecological security framework [17,18,19]. Located within the Hengduan Mountains—one of the world’s 36 biodiversity hotspots—the Upper Minjiang River Basin exhibits pronounced topographic complexity, high forest coverage, and a diverse assemblage of forest types [20]. These characteristics are shared by many mountain forest ecosystems worldwide, making the Upper Minjiang River Basin a representative case for investigating how forest-type heterogeneity influences ES supply and trade-off/synergy relationships under complex mountain environments and providing insights for sustainable forest management in similar mountain regions. This complex assemblage of forest types provides an important foundation for maintaining regional ESs, including water retention, carbon sequestration, soil conservation, and biodiversity maintenance. However, it may also lead to pronounced spatial disparities and mutual constraints among different ESs [19,21]. Based on these characteristics, this study proposes the following hypotheses: (1) ES supply and trade-off/synergy relationships differ significantly among forest types; (2) the strength and spatial distribution of ES trade-offs exhibit clear scale dependence and forest-type specificity; and (3) forest type, together with topographic factors, plays a dominant role in explaining the spatial differentiation of ES trade-offs.

## 2. Materials and Methods

### 2.1. Study Area

**Figure 1:**
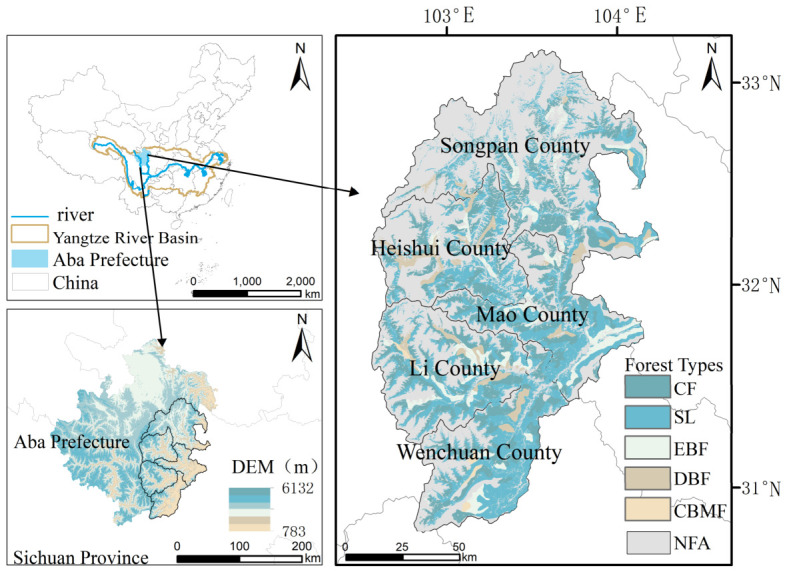
The location of the upper reaches of the Minjiang River, China. CF, coniferous forest; SL, shrubland; EBF, evergreen broad-leaved forest; DBF, deciduous broad-leaved forest; CBMF, coniferous and broad-leaved mixed forest; NFA, non-forest area; DEM, Digital Elevation Model.

The Upper Minjiang River Basin (102°32′–104°15′ E, 30°45′–33°09′ N), as shown in Figure 1, stretches longitudinally across the Minshan Mountains and the northwestern Sichuan Plateau. Originating from Gonggang Ridge of the Minshan Mountains, the river flows through the five county-level administrative regions of Songpan, Heishui, Maoxian, Lixian, and Wenchuan, covering an area of 24,800 km^2^, of which the forest coverage area is approximately 12,500 km^2^, making it one of the regions with the most abundant forest resources in the upper Yangtze River Basin. The vegetation zonation in this region is structurally complete. According to the classification system of Vegetation of China [22,23], a typical mountain gradient differentiation pattern is observed: the montane DBF belt occurs at elevations of 1600–2000 m, dominated by *Quercus aquifolioides, Betula albosinensis*, and Acer species, with distinct seasonal phenological changes; the CBMF belt at 2000–2600 m is composed of transitional communities formed by *Salix-Larix* and *Abies faxoniana-Betula albosinensis*, exhibiting complex species composition; the subalpine dark CF belt at 2600–3600 m is characterized by CF with *Abies faxoniana* and *Picea asperata* as the constructive species, representing the most extensive forest type and possessing the highest CS in the region. SL in this area primarily consists of woody vegetation at the pre-forest successional stage during forest community development, as well as dwarfed woody layers formed under constraints of high elevation or habitat limitations, rather than constituting an independent SL vegetation type [22].

### 2.2. Data Sources and Processing

This study compiled multi-source geospatial datasets for 2024, encompassing land use and digital elevation models. All datasets were harmonized through two preprocessing steps: first, projection into the WGS_1984_UTM coordinate system; second, resampling to a uniform spatial resolution of 30 m × 30 m. Such standardization ensures consistency across subsequent model computations and spatial analyses. Detailed information regarding data provenance is provided in Table 1.

### 2.3. Methodologies for Assessing ESs

By integrating multiple modules within the InVEST (v3.14.3) model, this study quantified five key ESs in the Upper Minjiang River Basin. This unified approach improves the comparability and interpretability of the results across different services.

#### 2.3.1. Carbon Storage

The module first performs localized calibration of the four carbon pool parameters for different forest types based on published carbon density literature and existing regional studies [24,25,26,27,28].

C_total_ = C_above_ + C_below_ + C_soil_ + C_dead_ (1)

#### 2.3.2. Soil Conservation

In this part, we utilized the InVEST sediment retention module to evaluate this function [29,30,31,32,33,34], with relevant parameters presented in Table S2:

RKLS = R × K × L × S(2)

ULSE = R × K × L × S × C × P(3)

SR = RKLS − ULSE(4)

RKLS is potential soil erosion (t/hm^2^), ULSE is actual soil erosion (t/hm^2^), and SR signifies the soil retention quantity (t/hm^2^), which is calculated as the difference between potential and actual soil erosion. R represents the rainfall erosivity factor, K represents the soil erodibility factor, LS indicates the topographic factor, C refers to the cover and management factor, P corresponds to the support practice factor.

#### 2.3.3. Water Yield

This module quantifies watershed water yield by balancing precipitation against losses from evapotranspiration, canopy interception, and soil moisture retention. The Zhang coefficient (Z) was set to 4.433 [35], while the Kc (plant evapotranspiration coefficient) and maximum root depth were calibrated based on references [36,37,38,39] and adjusted according to forest vegetation characteristics. The involved calculation formulas are as follows, with relevant parameters presented in Table S3:

WY = (1 − AET/Q) Q(5)

W_total_ = Y/1000 × A (6)

WY represents water yield capacity; Q is annual precipitation (mm); Wtotal is total water yield (m^3^); Y is average water yield depth (mm); and A is watershed area (m^2^).

#### 2.3.4. Habitat Quality

This module is constructed based on land-use stress intensity and habitat suitability, and is used to characterize biodiversity maintenance capacity. Higher habitat quality index values in the module indicate richer regional biodiversity, better integrity, lower fragmentation, and less human activity impact [40,41,42], with relevant parameters presented in Tables S4 and S5:

(7) $$HQ_{i}\;=\;H_{i}\left[1-\left(\frac{D_{i}^{z}}{D_{i}^{z}+k^{z}}\right)\right]$$

In Equation (5), H_i_ corresponds to the suitability score assigned to land-use category i; D_xj_ is the cumulative stress intensity from anthropogenic disturbances; Z represents the shape parameter (0.05); K refers to the half-saturation coefficient (0.5); HQ_i_ represents habitat quality.

#### 2.3.5. Forest Stock Volume

Forest stock volume is an important indicator connecting forest resource structure and CS. This study initially employed the CASA (v1.0) to quantify vegetation net primary productivity (NPP). On this basis, referring to existing studies, NPP, biomass, and stock volume conversions were established to convert NPP into aboveground biomass, and forest stock volume was then estimated by combining stock volume conversion coefficients for different forest types [43,44,45,46]. The conversion formulas are presented in Table S6.

### 2.4. Methods for Analyzing ES Trade-Offs and Synergies

#### 2.4.1. Spearman Correlation Analysis

Due to the non-normal distribution observed in the data (Shapiro-Wilk test, *p* < 0.05), this research utilized Spearman rank correlation analysis to examine the patterns of trade-offs and synergies among ESs across various forest types. The correlation coefficient ranges from −1 to 1, where a value of 1 signifies a complete positive monotonic relationship, −1 denotes a complete negative monotonic relationship, and 0 indicates an absence of monotonic association; a greater absolute value of the coefficient reflects a stronger correlation. After conducting significance testing, a *p*-value of less than 0.05 was deemed to indicate a significant trade-off or synergy effect among ESs. The specific formula is presented as follows:

(8) $$\begin{array}{c} \rho =\frac{\sum_{i=1}^{n}\left(R_{x_{i}}-{\bar{R}}_{x}\right)\left(R_{y_{i}}-{\bar{R}}_{y}\right)}{\sqrt{\sum_{i=1}^{n}{\left(R_{x_{i}}-{\bar{R}}_{x}\right)}^{2}\sum_{i=1}^{n}{\left(R_{y_{i}}-{\bar{R}}_{y}\right)}^{2}}} \end{array}$$

The Spearman rank correlation coefficient, denoted as ρ, is calculated as follows: n refers to the total observation count; $R_{x_{i}}$ and $R_{y_{i}}$ refer to the rank positions of the i-th observation values for variables x and y; ${\bar{R}}_{x}$ and ${\bar{R}}_{y}$ signify the averaged rankings of the two variables.

#### 2.4.2. Root Mean Square Deviation

This study employed the RMSD index to uniformly standardize each service and measure the degree of conflict [47]:

(9) $$RMSD=\sqrt{\frac{1}{n}\sum_{i=1}^{n}{\left(V-{\hat{V}}_{i}\right)}^{2}}$$

In this formula, n represents the total number of observations; ${\hat{V}}_{i}$ represents the mean standardized ES value; and V_i_ is the standardized value of the ith observation. The trade-off intensity between paired services is assessed by their deviation from the diagonal line y = x: values falling on the diagonal indicate no trade-off, proximity to the diagonal indicates a smaller trade-off value, and distance from the diagonal indicates a larger trade-off value.

### 2.5. Driving Mechanism Analysis Methods

#### 2.5.1. Factor Detector

This study identified seven key environmental and socioeconomic variables as determinants influencing forest ESs in the Upper Minjiang River Basin, and performed spatial discretization processing on these variables: DEM [36,48], slope, precipitation [36], distance to roads, population density [36,49], and the distance to hydropower stations [50,51]. The GeoDetector model [52] was utilized to investigate the underlying mechanisms of ES trade-offs. The dependent variable (Y) was defined as the pairwise trade-off magnitude among ESs, with seven potential drivers (X) being fed into the model to parse their individual and interactive influences on the geographic variation in trade-off intensity.

#### 2.5.2. Interaction Detector

Utilizing the interaction detector, we evaluated the effects of two driving factors on the trade-offs of ES and investigated whether their joint influence strengthens, diminishes, or affects spatial variations in trade-off relationships among ESs in a nonlinear manner.

### 2.6. ES Bundle Identification

ES bundles were identified using a spatially constrained K-means clustering approach. In addition to minimizing the distance between observations and cluster centroids, the method incorporates spatial adjacency constraints to account for the spatial relationships among neighboring units. Consequently, the resulting ES bundles are spatially contiguous, thereby reducing the fragmentation typically observed in conventional K-means clustering results [53,54,55].

(10) $$J_{min}={\sum_{k=1}}^{K}[\sum_{xi}\in C_{k}{\left\|x_{i}-\mu_{k}\right\|}^{2}+\lambda \cdot S(C_{k})]$$

(11) $$S(C_{k})=\sum_{xi,xj}\in C_{k,i}\neq_{\;j}(1-w_{\mathrm{ij}})$$

J_min_-Objective function of the spatially constrained K-means (SCK-means) clustering algorithm; K-Number of clusters; C_k_-The k-th cluster; x_i_-The i-th data point; μ_k_-Centroid of the k-th cluster; λ-Spatial constraint weighting parameter; S(C_k_)-Spatial connectivity penalty term; w_ij_-Spatial adjacency weight between data point i and data point j.

**Figure 2:**
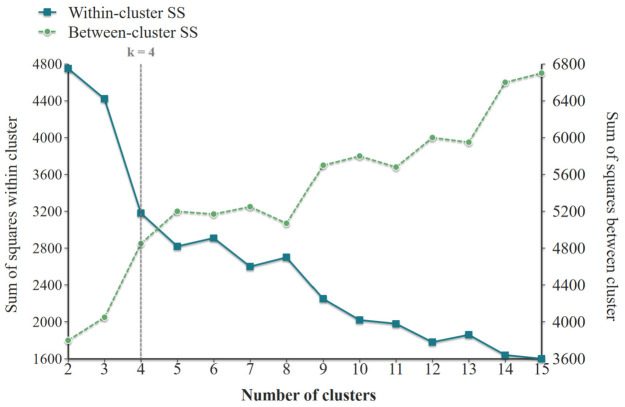
Identification of the optimal cluster number for ES bundles. WCSS quantifies how tightly grouped the samples are within each ES bundle; BCSS measures how distinct the bundles are from one another—together they determine the optimal clustering solution for management-oriented functional zoning.

To eliminate the arbitrariness involved in selecting the ideal cluster number for SCK-means and to suppress data fluctuations, ES bundles were classified according to the mean magnitudes of various ESs. A grid of 1000 × 1000 m was generated as the basic analysis unit. As shown in Figure 2, clusters were defined with a range from 2 to 15, and the within-cluster sum of squares (WCSS) and between-cluster sum of squares (BCSS) were calculated for each clustering scenario. The within-cluster sum of squares (WSS) assessed the similarity of samples within each ES bundle, with lower values indicating higher within-cluster homogeneity. The between-cluster sum of squares (BSS) evaluated the differences among ES bundles, with higher values indicating greater inter-cluster heterogeneity. The results showed that the WSS decreased substantially as the number of clusters increased to k = 4, whereas the rate of decline became less pronounced with further increases in cluster number. Meanwhile, the BSS continued to increase after k = 4, indicating an improved separation among clusters. Therefore, k = 4 was considered to provide an optimal balance between within-cluster homogeneity and between-cluster heterogeneity. Accordingly, four ES bundles were identified and subsequently used to delineate four forest management zones.

### 2.7. Technical Framework

**Figure 3:**
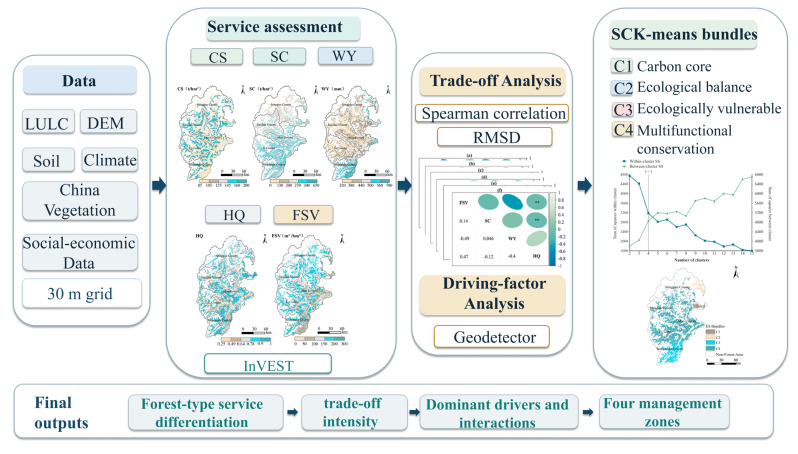
Technical Framework of the study. CS-Carbon Storage; SCSoil Conservation; WY-Water Yield; HQ-Habitat Quality; FSV-Forest Stock Volume; RMSD-root mean square deviation.

As shown in Figure 3, the technical framework of this study comprises four main components. First, input data-including DEM, land use, soil, climate, vegetation, and socio-economic data-were prepared at a 30 m grid resolution. Second, five key ESs (CS, SC, WY, HQ, and FSV) were quantified using the InVEST model. Third, trade-offs and synergies among these services were assessed through Spearman correlation and RMSD, and the driving mechanisms were identified using GeoDetector. Finally, SCK-means clustering was applied to delineate ES bundles and translate them into four spatial management zones, supporting differentiated forest management strategies.

## 3. Results

### 3.1. Spatial Heterogeneity of ESs

#### 3.1.1. Supply Patterns of Forest ESs at the Regional Scale

**Figure 4:**
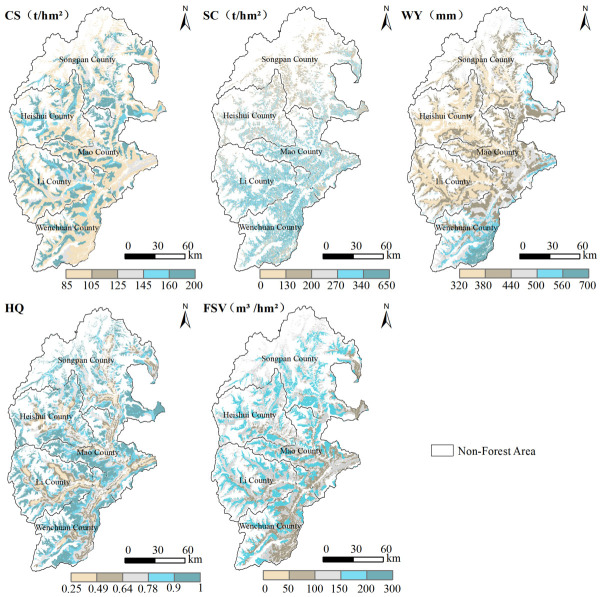
Spatial distribution of ESs. CS-carbon storage; SC-soil conservation; WY-water yield; HQ-habitat quality; FSV-forest stock volume.

The Upper Minjiang River Basin forest ecosystem displayed marked spatial variability in ES distribution and intensity, as shown in Figure 4. In CS, the region reached 1.78 × 10^8^ t, averaging a density of 141.91 t/hm^2^. The SC data was also notable, with total amounts hitting 2.98 × 10^8^ t. This brings the mean retention capacity to 242.29 t/hm^2^, while keeping the soil erosion modulus low at 7.09 t/hm^2^. For WY, we recorded a total yield of 6.48 × 10^9^ m^3^, which translates to a mean water depth of 522.53 mm. Meanwhile, the environment maintained a solid habitat quality index of 0.78. Lastly, forest stock volume capped at 1.20 × 10^8^ m^3^, leaving the region with a steady average of 95.71 m^3^/hm^2^.

#### 3.1.2. Differences in ES Supply Among Forest Types

**Figure 5:**
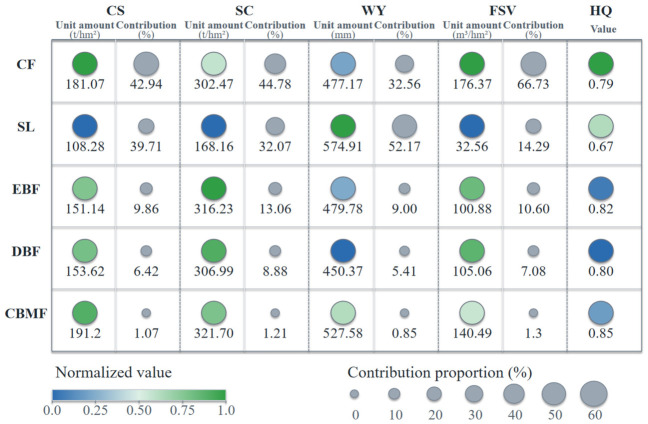
Comparison of ESs among different forest types. CS, carbon storage; SC, soil conservation; WY, water yield; HQ, habitat quality; FSV, forest stock volume; CF, coniferous forest; SL, shrubland; EBF, evergreen broad-leaved forest; DBF, deciduous broad-leaved forest; CBMF, coniferous and broad-leaved mixed forest.

As shown in Figure 5, different forest types vary significantly in their ES provision.

Among all forest types, CF performed remarkably well in CS, FSV, and SC. Specifically, their carbon density reached 181.07 t/hm^2^, and stock volume hit 176.37 m^3^/hm^2^. Soil conservation in CF also exceeded that of other types. Despite lower per-unit ES levels, SL played a dominant role in regional water supply. This was largely due to their extensive spatial coverage, which enabled them to contribute 52.17% of total water yield and thus serve as a key source of runoff generation. Evergreen broad-leaved forests (EBF) and DBF showed intermediate levels of ESs. EBF remained relatively stable in WY, while DBF showed higher values in CS and SC compared with EBF and SL. However, their total contributions were lower than CF due to smaller spatial extent. CBMF exhibited extremely high values per unit, but due to their very limited distribution range in the region, their actual contribution to the total ESs of the entire area is minimal.

### 3.2. Analysis of Trade-Offs and Synergies Among ESs

#### 3.2.1. Correlation Analysis of Trade-Offs and Synergies Among ESs

1. Correlation Analysis of ESs at the Regional Scale

**Figure 6:**
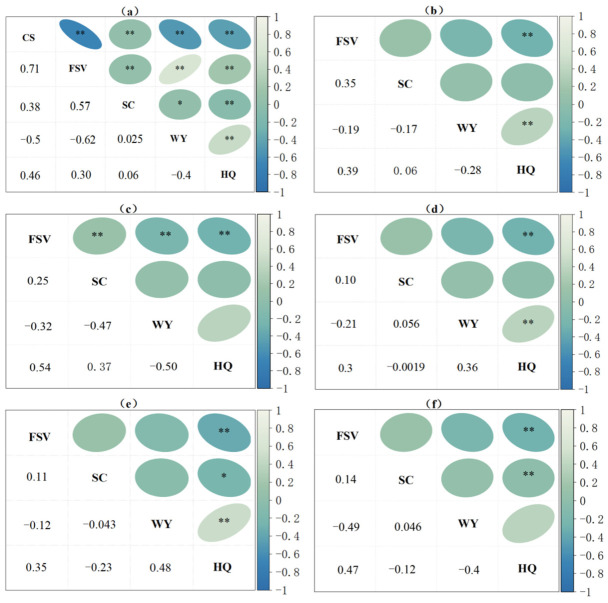
Correlation analysis of ESs. (**a**) Regional scale; (**b**) CF, coniferous forest; (**c**) SL, shrubland; (**d**) EBF, evergreen broad-leaved forest; (**e**) DBF, deciduous broad-leaved forest; (**f**) CBMF, coniferous and broad-leaved mixed forest; ** denotes *p* < 0.01 and * denotes *p* < 0.05.

At the regional scale, ESs in the upper Minjiang River forests showed both synergies and trade-offs (Figure 6a). CS and FSV exhibited the strongest positive correlation (r = 0.71, *p* < 0.01). FSV was also positively correlated with SC, and CS with HQ. Trade-offs were mainly observed between WY and other services. The correlations for CS-WY, FSV-WY, and WY-HQ were all significantly negative (*p* < 0.01).

2. Correlation Analysis of ESs at the Forest Type Scale

At the forest-type scale, ES relationships exhibited pronounced heterogeneity (Figure 6b–f). Overall, correlation strengths were weaker than those observed at the regional scale, and some relationships even reversed direction. In CF and SL, FSV-HQ and FSV-SC showed significant positive correlations, whereas WY was generally negatively correlated with HQ and SC, highlighting strong trade-offs associated with hydrological services. In EBF, relationships among ESs were generally weak, with only WY-HQ exhibiting a positive correlation. In DBFs, HQ was positively correlated with WY, indicating strong synergistic effects. CBMF displayed the most pronounced trade-offs: WY-HQ showed a significant negative correlation, whereas HQ-FSV exhibited a strong positive correlation.

#### 3.2.2. Analysis of Trade-Off and Synergy Intensity of ESs

1. Analysis of Trade-off Intensity at the Regional Scale

**Figure 7:**
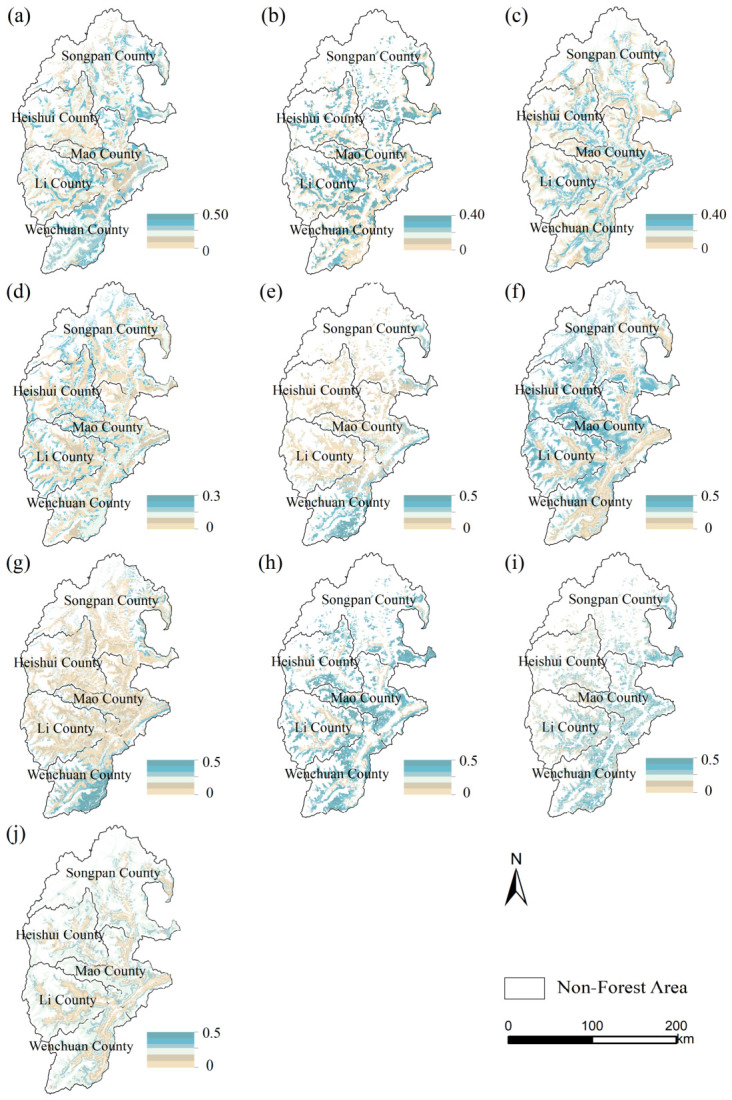
Trade-off intensity between pairwise ESs at the regional scale. (**a**) CS-WY; (**b**) CS-SC; (**c**) CS-HQ; (**d**) CS-FSV; (**e**) WY-SC; (**f**) WY-HQ; (**g**) WY-FSV; (**h**) SC-HQ; (**i**) SC-FSV; (**j**) HQ-FSV.

At the regional scale, trade-off patterns were primarily concentrated among water-yield-related ES pairs and exhibited pronounced spatial clustering characteristics (Figure 7). CS-WY, WY-HQ, WY-FSV, HQ-FSV, and SC-HQ were the major service pairs with comparatively elevated trade-off intensity. The areas of high value were primarily located in the mountainous regions of Mao County, Li County, and Wenchuan County, spanning the central and southern parts of the basin. In contrast, lower trade-off intensities were noted in northern Songpan County and adjacent non-forest regions. The spatial patterns of different services vary significantly. Specifically, the principal distribution zones of CS-SC and SC-HQ are distributed in a banded pattern along mountainous regions, exhibiting a strong topographic dependence. The principal distribution zones of WY-FSV and HQ-FSV were predominantly concentrated in the central and southern regions of the basin, showing a notable degree of spatial continuity, whereas CS-FSV and WY-SC demonstrated generally weaker trade-off intensities with more dispersed spatial distributions.

2. Analysis of Trade-off Intensity of ESs at the Forest Type Scale

**Figure 8:**
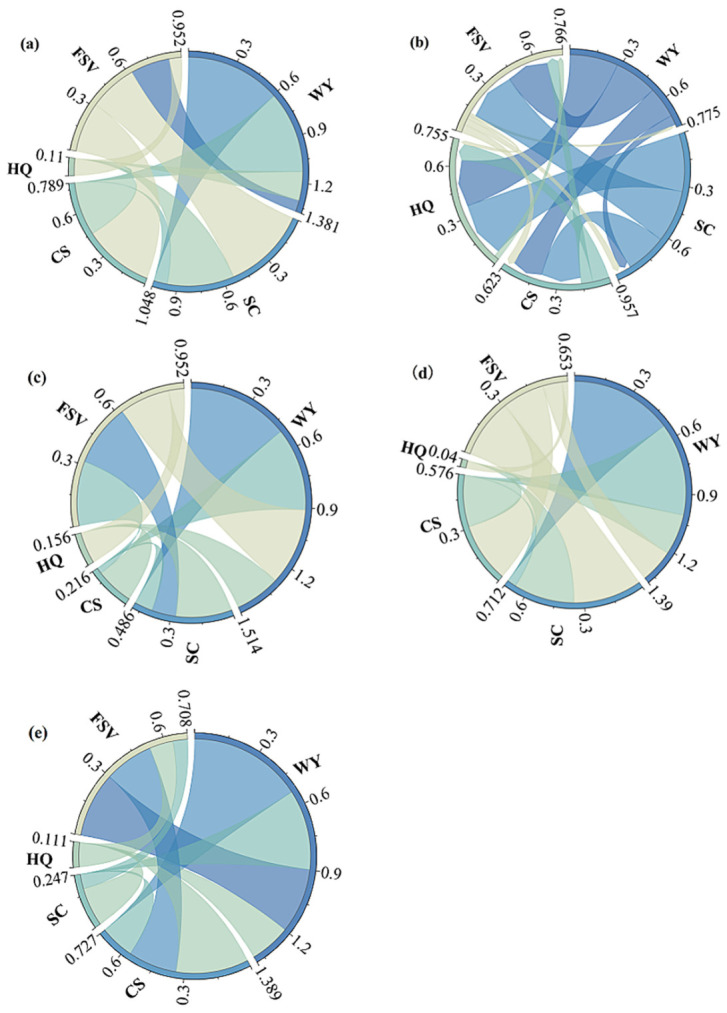
Trade-off intensity between pairwise ESs at the forest type scale. (**a**) CF, coniferous forest; (**b**) SL, shrubland; (**c**) EBF, evergreen broad-leaved forest; (**d**) DBF, deciduous broad-leaved forest; (**e**) CBMF, coniferous and broad-leaved mixed forest; CS, carbon storage; SC, soil conservation; WY, water yield; HQ, habitat quality; FSV, forest stock volume.

At the scale, different forest ecosystems exhibited considerable variation in the magnitude of ES trade-offs (Figure 8).

The trade-off patterns differed markedly across forest types. In CF (a), WY registered the highest trade-off intensity at 1.381, driven mainly by its conflicts with CS and FSV. SL (b) presented a more balanced structure, with no single service dominating; WY here showed a moderate intensity of 0.775. EBF (c), by contrast, displayed a strongly WY-centered pattern, where the trade-off intensity reached 1.514-substantially exceeding that of all other services. Similarly, DBF (d) and CBMF (e) also showed WY-dominated trade-off structures, with intensities of 1.390 and 1.389, respectively.

### 3.3. Analysis of Driving Mechanisms of ES Trade-Offs

#### 3.3.1. Single-Factor Detection Results

**Figure 9:**
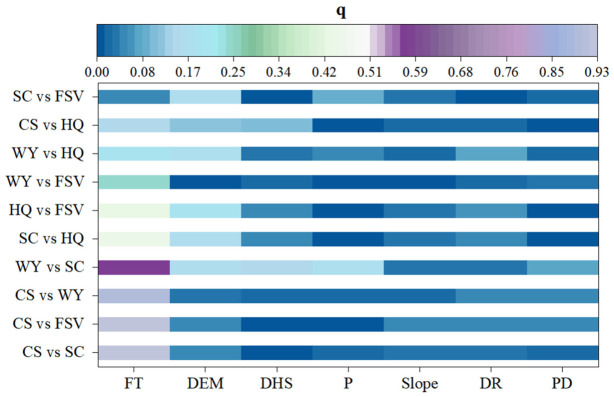
Contribution rates of driving factors to trade-off relationships between pairwise ES; FT-forest type; DHS-distance to hydropower station; P-precipitation; DR-distance to road; PD-population density; DEM-Digital Elevation Model.

GeoDetector results revealed substantial differences in the explanatory power of individual driving factors on ES trade-off relationships, exhibiting an overall pattern characterized by forest type dominance, secondary control by topographic factors, and auxiliary regulation by other environmental and socioeconomic variables. As shown in Figure 9, forest type emerged as the strongest explanatory factor across all trade-off relationships. This dominance was especially pronounced for CS-WY, CS-SC, and CS-FSV pairs, where forest type consistently exhibited the highest q values. In correlation analysis, precipitation and elevation gained slightly more influence, yet forest type remained dominant. For the WY-FSV relationship, all factors showed generally low explanatory power; only forest type showed a relatively noticeable impact here, suggesting that this service pair is less sensitive to environmental changes.

#### 3.3.2. Multi-Factor Interactive Driving Effects

The interaction detector showed that all factor pairs produced enhancement effects, and non-linear enhancement was the main type. Among all pairs, forest type worked best with other factors. Whether paired with terrain or climate factors, its combined explanatory power rose markedly.

In carbon-storage-related trade-offs, the interplay between forest type and other variables proved the most influential, with its explanatory power remaining at high levels across all factor combinations, exhibiting two-factor enhancement effects, whereas pairwise interactions among other factors were predominantly non-linear enhancement, indicating that forest type still occupies a dominant position in the interaction process. In hydrological-service-related relationships, interactions were mainly characterized by non-linear enhancement. In habitat-quality-related relationships, interactions were more complex, with most factor combinations—such as elevation ∩ distance to roads and forest type ∩ elevation—demonstrating comparatively elevated explanatory power.

### 3.4. Spatial Distribution of ES Bundles

**Figure 10:**
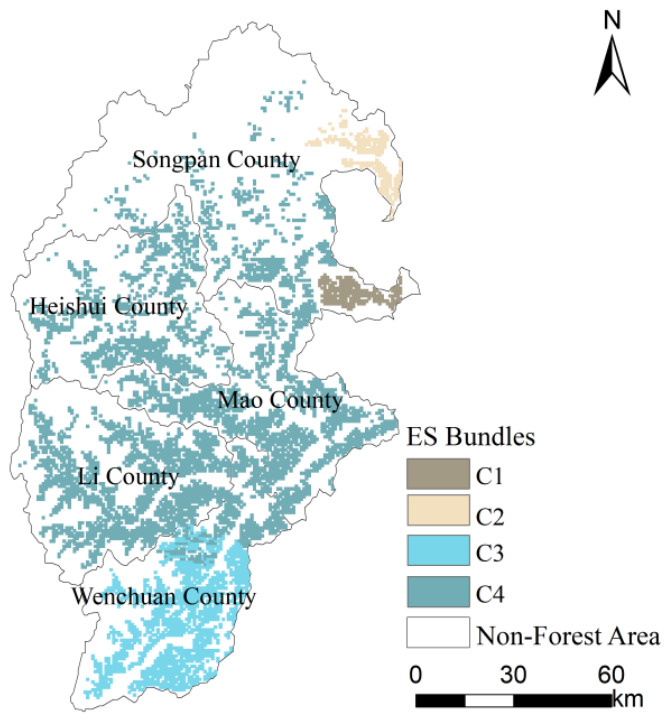
The result of ES bundles. C1, crbon storage core zone; C2, ecological balance zone; C3, ecologically fragile zone; C4, multifunctional conservation zone.

Drawing upon the SCK-means algorithm with the cluster count fixed at 4, the spatial configuration of ES bundles throughout the study domain was derived through iterative optimization. The characteristics of regional ecological functions are reflected through the names of functional zones; as shown in Figure 10, this study named the four zones as the carbon sequestration core zone, ecological balance zone, ecologically fragile zone, and multifunctional conservation zone according to their ecological functional characteristics.

#### 3.4.1. Carbon Sequestration Core Zone

The carbon sequestration core zone covers an area of 50,725.44 hm^2^, comprising 4.05% of the overall research domain, making it the smallest ES bundle in terms of area. It is mainly distributed in the border region between southeastern Songpan County and northern Mao County. This zone is mainly covered by SL (40.91%) and CF (37.48%). EBF and DBF together make up 21.61%. The standardized value of CS is the highest (0.620). The other services—SC, WY, and FSV—are all low, at 0.301, 0.174, and 0.253 respectively.

#### 3.4.2. Ecological Balance Zone

The ecological balance zone covers an area of 61,270.02 hm^2^, accounting for 4.89% of the total study area, and is mainly located in the northeastern part of Songpan County. This zone is dominated by SL (45.72%) and CF (37.72%). The standardized values of the ESs are relatively balanced among CS, WY, and FSV (0.497, 0.351, and 0.304, respectively).

#### 3.4.3. Ecologically Fragile Zone

The area identified as ecologically sensitive spans 264,027.43 hm^2^, which represents 21.09% of the overall research region, primarily situated in Wenchuan County. This zone is dominated by SL (58.08%). CS and HQ are 0.538 and 0.699, respectively. SC and WY are 0.036 and 0.204, respectively.

#### 3.4.4. Multifunctional Conservation Zone

The multifunctional conservation zone covers an area of 875,951.51 hm^2^, comprising 69.96% of the overall research domain, making it the largest ES bundle in terms of area. This zone has the richest forest composition, dominated by SL (43.39%) and CF (38.43%). WY (0.464), HQ (0.758), and FSV (0.306) all reach the highest levels, while CS (0.410) and SC (0.049) are at intermediate levels.

## 4. Discussion

### 4.1. Regulatory Effects of Forest Type Heterogeneity on ES Provision

The forest ecosystem in the Upper Minjiang River Basin exhibits a strong capacity for multifunctional ES provision, particularly in WY, SC, and HQ. While this aligns with previous regional assessments of water conservation and ES value [30,35], this study advances beyond aggregate-level descriptions by revealing how internal forest-type heterogeneity shapes service supply patterns. Rather than treating forest ecosystems as functionally uniform entities, we demonstrate that distinct combinations of CS, WY, SC, HQ, and FSV emerge from differences in stand structure, canopy morphology, root architecture, biomass accumulation, and successional stage.

Carbon–water trade-offs in CF is significant. High CS in CF is structured by stand-level attributes: large aboveground biomass, extended stand age, and suppressed decomposition under low-temperature subalpine conditions prolong carbon residence time. However, this carbon sink advantage incurs a hydrological cost. The thick litter layer and extensive root system that enhance soil carbon stabilization simultaneously increase water interception and evapotranspiration loss [56]; under cold subalpine conditions, slow litter decomposition leads to persistent coarse organic matter accumulation, forming an efficient “biological buffer zone” for soil conservation—yet this very structural advantage intensifies the carbon–water trade-off. Higher biomass inevitably drives greater evapotranspiration and canopy interception losses according to watershed water balance principles. Sun [38] confirmed that WY declines significantly as forest carbon density increases at the catchment scale, but could not identify how this trade-off differs among forest types. Our study advances this by scaling the pattern down to the forest-type level, identifying CF as the functional group where carbon–water tension is most pronounced—its per-unit-area CS and SC significantly exceed other forest types, but at a measurable cost to WY. This implies that CF expansion in water-source protection zones may compromise water yield, whereas in carbon-sequestration priority areas, CF remains irreplaceable. Such context-dependent, forest-type-specific mechanisms correct “one-size-fits-all” management strategies.

### 4.2. Trade-Off Mechanisms of ES Relationships

At both regional and forest-type scales, significant synergistic relationships between CS and FSV were observed, whereas distinct trade-off characteristics with WY were evident.

Both CS and FSV are directly controlled by forest biomass accumulation; stands with high FSV typically have larger diameter at breast height, higher canopy coverage, and leaf area index, enabling them to fix more atmospheric carbon through photosynthesis, thereby forming higher CS capacity [32]. Therefore, the two showed a stable synergistic relationship. However, increased CS did not necessarily result in higher WY capacity. High CS areas in the Upper Minjiang River Basin were mostly found in high-elevation dark CF and mature conifer-dominated regions. These forests have high biomass and carbon storage capacity. But their well-developed canopy structures change how precipitation is split up. According to the watershed water balance principle, water yield represents the portion of precipitation remaining after evapotranspiration losses. So when forests have more biomass, they lose more water through evapotranspiration, and that leaves less water for the region. Jackson [57], based on global-scale studies, pointed out that forest carbon sink get stronger, watershed WY capacity often drops. This is especially clear in areas where high-biomass forests are expanding. Liu [58] also found, in the Minjiang River Basin, that ecosystem productivity and water yield are negatively correlated.

### 4.3. Driving Mechanisms of Service Trade-Off Relationships

Existing studies have shown that forest structural attributes are important factors explaining the provision of forest ESs and their trade-off and synergy relationships, and their effects even exceed those of environmental factors in some cases [10].

In addition, topographic factors such as DEM and slope exhibited relatively high explanatory power, suggesting that mountain environmental gradients constitute an important foundation driving the spatial differentiation of ESs [16]. As a typical mountain ecosystem situated at the transitional belt from the eastern margin of the Qinghai–Tibet Plateau to the Sichuan Basin, the Upper Minjiang River Basin exhibits significant elevation gradients that lead to systematic changes in temperature, hydrothermal conditions, and vegetation composition, thereby affecting the supply capacity of ESs such as CS, WY, SC, and HQ. This is consistent with previous findings that identify elevation as an important environmental factor shaping trade-off and synergy patterns of ESs in mountain ecosystems [37,59]. Slope plays a key role by regulating runoff generation, soil erosion, and water infiltration, thereby influencing the redistribution of soil organic carbon, nitrogen, and other nutrients. These processes affect vegetation growth, biomass accumulation, and soil conservation capacity, ultimately contributing to spatial variations in carbon storage, water yield, and soil conservation. Recent studies have similarly highlighted that slope exerts an important influence on soil carbon, nitrogen dynamics, and nutrient redistribution in mountain ecosystems, further supporting its role as a key environmental driver of ecosystem functioning [60].

Besides natural environmental factors, the GeoDetector results also indicated that anthropogenic variables (distance to hydropower stations, roads, and population density) also contributed to ES trade-off differentiation, though with lower explanatory power than forest type and topography. Human activities alter ES relationships by modifying vegetation conditions, landscape connectivity, and ecological processes [61,62]. Although the Upper Minjiang River Basin remains a nationally important ecological functional zone with relatively limited human disturbance, localized anthropogenic activities may still influence ES interactions. Therefore, effective ecosystem management should simultaneously consider the combined effects of forest type, environmental gradients, and human activities to promote the coordinated enhancement of multiple ESs.

### 4.4. Zoning Management Strategies Based on ES Bundles

Compared with optimizing a single service, ES bundles offer a more effective framework. As Raudsepp-Hearne [47] demonstrated, bundling translates complex trade-off and synergy relationships into spatially explicit management units and captures the co-occurrence patterns of multiple ESs. Building on this, Kline [63] further showed that applying the ecosystem services concept to public land management supports differentiated strategies based on dominant ecological functions. Accordingly, we delineated four functional zones as follows.

The carbon sequestration core zone is characterized by high CS, but WY and SC there fall short. The primary risk is that a singular pursuit of carbon sinks may exacerbate functional deficiencies in water and soil regulation. To address this, the management objective should focus on enhancing forest structural diversity and water–soil regulation capacity while protecting high-carbon-storage stands. Specifically, following what Bauhus [64] advocated, transitioning to a retention approach through near-natural management, moderate tending, and gap regulation can promote the formation of CBMF structures, thereby reconciling carbon retention with hydrological and soil functions.

The ecological equilibrium zone exhibits relatively balanced multiple services, with ecological risks primarily arising from external disturbances that may disrupt existing synergy patterns. Drawing on Lindenmayer [65], the management core of this zone lies in reducing large-scale artificial hard interventions to conform to and accelerate community succession patterns. In parallel, Dai [46] showed that management intensification significantly affects multiple ecosystem services; therefore, policy orientation should adopt guided natural succession strategies rather than intensive silviculture, focusing on protecting understory regeneration layers and utilizing the self-driven succession mechanisms of plant communities toward climax communities to construct multi-species, interlaced, structurally complex, and stress-resistant forest ecosystem ecological barriers.

The multifunctional conservation zone faces high human disturbance, requiring dual ecological and socioeconomic functions. Musavandalo [61] and Solomon [62] demonstrated that human disturbances reduce carbon stocks, with effects mediated by elevation, patch size, and connectivity. In the UMRB, this pattern is reflected in the spatial distribution: high-carbon CF concentrates in higher-elevation zones with lower disturbance, whereas low-efficiency patches near residential areas suffer biomass carbon losses. Thus, management must spatially match forest-type configurations to disturbance regimes. A “nested patch” configuration interweaving high-WY SL, high-CS CF, and high-HQ CBMF should be adopted to utilize edge effects and functional complementarity. For low-efficiency patches near residential areas, non-timber forest products or near-natural wellness forests should be developed without destroying stand structure, aligning with Gao [66] on integrating ES valuation with socioeconomic development.

### 4.5. Research Uncertainties and Future Prospects

This study has several limitations that should be acknowledged. First, the ESs were quantified using model-based approaches, and uncertainties associated with model parameters and input datasets may have influenced the estimation results. Second, although parameter values were selected from published studies conducted in ecologically similar regions, field observations were not available for direct validation of the modeled ESs. Finally, this study represents a static assessment based on a single period and therefore does not capture the temporal dynamics of ESs or their trade-off relationships under climate change and human disturbances. Future research should integrate long-term monitoring data, field measurements, and dynamic analyses to further improve the reliability and applicability of ES assessments.

## 5. Conclusions

This study demonstrates that the supply and interactions of forest ESs in the Upper Minjiang River Basin are strongly associated with forest type and exhibit clear spatial differentiation along elevation gradients. CF contribute most to CS and FSV, whereas SL play a particularly important role in sustaining watershed WY.

ES relationships exhibit pronounced scale dependence and forest-type differences. At the regional scale, CS and FSV show strong synergies, while both have clear trade-offs with WY. At the forest-type scale, WY is frequently involved in trade-offs across most forest types, and it acts as a key node in the trade-off network, followed by SC and FSV, while HQ shows relatively low participation.

GeoDetector analysis revealed a clear hierarchy among the drivers of ES trade-offs. Forest type exhibited the highest explanatory power and was identified as the key factor associated with the spatial differentiation of ES trade-offs, while topographic variables, particularly elevation and slope, also played important roles. In addition, human-related factors, including distance to hydropower stations, distance to roads, and population density, contributed to the observed spatial patterns, although their explanatory power was generally lower.

Management priorities should vary across zones. In the carbon sequestration core zone, the focus ought to be on preserving carbon sinks and refining stand structure. For the ecologically fragile zone, efforts should center on boosting soil conservation and regulating water flows. The ecological balance zone calls for sustaining a balance among multiple functions. Meanwhile, the multifunctional conservation zone needs to enhance water retention, maintain habitat connectivity, and implement ecological compensation.

## Figures and Tables

**Table 1:** Dataset provenance and technical specifications employed in this research.

Data	Type/Resolution	Data Source	Website
CLCD	Raster/30 m	CLCD	https://doi.org/10.5281/zenodo.15853565
DEM	Raster/30 m	Geospatial Data Cloud	https://www.gscloud.cn
China vegetation	Vector	Resource and Environmental Science and Data Center, CAS	https://www.resdc.cn/
Evapotranspiration	Raster/500 m		
Precipitation	Raster/1000 m	National Tibetan Plateau Data Center	https://data.tpdc.ac.cn
Temperature dataset	Raster/1000 m		
Soil data	Raster/1000 m		https://cds.climate.copernicus.eu
MODIS NDVI	Raster/250 m	NASA LP DAAC	https://lpdaac.usgs.gov/products/mod13q1v061/ (accessed on 20 January 2026)
Solar radiation	Raster/1000 m	ERA5-LAND	https://data.tpdc.ac.cn
Road data	Vector	National Bureau of Statistics	http://www.stats.gov.cn
Population density	Raster/1000 m	Resource and Environmental Science and Data Center, CAS	https://www.resdc.cn/
Hydropower station	Point	Global Hydropower and Reservoir Dataset	https://doi.org/10.5281/zenodo.14526360

## Data Availability

The datasets used and/or analyzed during the current study are available from the corresponding author upon reasonable request.

## Supplementary Materials

The following supporting information can be downloaded at https://www.mdpi.com/article/10.3390/plants15142149/s1, Table S1: Carbon pools; Table S2: Biophysical table for water yield; Table S3: Biophysical table for soil conservation; Table S4: Sensitivity of each land use type to threat factors; Table S5: Threat factor properties; Table S6: Conversion formulas between NPP and the wood volume for different forests; Table S7: The result of interaction detector.
