# Supplementary material for: Trade-Offs and Synergies Among Ecosystem Services Influenced by Forest Type and Their Implications for Spatial Management in the Upper Minjiang River Basin, China
Source: Plants (Basel). 2026 Jul 12;15(14):2149. doi: 10.3390/plants15142149 (PMC13414793; doi:10.3390/plants15142149)
Supplement: Supplementary file 1 [file plants-15-02149-s001.zip › plants-4404161-supplementary.pdf]

Supplementary Materials

Table.S1 Carbon pools.

Lucode	LULC_name	C_above	C_below	C_soil	C_dead
1	Coniferous Forest	46.67	9.334	122.1	2.97
2	Shrubland	8.1	2.56	96.7	0.92
3	Evergreen Broad-leaved Forest	38.83	7.76	102.03	2.52
4	Deciduous broad-leaved Forest	39.69	7.94	103.7	2.29
5	Mixed Forest	51.4	10.28	125.6	3.92

Table.S2 Biophysical table for water yield.

Description	Root_depth	Kc	LULC_veg
Coniferous Forest	6000	1	1
Shrubland	3000	0.5	1
Evergreen Broad-leaved Forest	4900	1	1
Deciduous broad-leaved Forest	4900	1	1
Mixed Forest	6300	1	1

Table.S3 Biophysical table for soil conservation.

Description	Lucode	Usle_c	Usle_p
Coniferous Forest	1	0.01	1
Shrubland	2	0.05	1
Evergreen Broad-leaved Forest	3	0.02	1
Deciduous broad-leaved Forest	4	0.02	1
Mixed Forest	5	0.01	1

Table.S4 Sensitivity of each land use type to threat factors.

LULC	LULC use type	Habitat	Gendi	Butoushuimian	Road
1	Coniferous Forest	1	0.6	0.9	0.8
2	Shrubland	0.8	0.5	0.8	0.7
3	Evergreen Broad-leaved Forest	0.9	0.7	0.9	0.8
4	Deciduous broad-leaved Forest	0.9	0.7	0.9	0.8
5	Mixed Forest	0.95	0.8	1	0.9

Table.S5 Threat factor properties.

MAX_DIST	Weight	Threat	DECAY
5	0.7	Gendi	linear
10	1	Butoushuimian	exponential
8	0.8	Rroad	linear

Table.S6 Conversion formulas between NPP and the wood volume for different forests

Forest Type	Conversion formula
Coniferous Forest	V=(y/2.98)^6.71
Shrubland	V=[1.18(y−12.8)−0.72]^0.81
Evergreen Broad-leaved Forest	V=(y−3.50)/0.0323
Deciduous broad-leaved Forest	V=(y−3.50)/0.0323
Mixed Forest	V=((y/2.98)^6.71+(y−3.50)/0.0323)/2

Table.S7 The result of interaction detector (FT-Forest Type，DHS-Distance to Hydropower Station，P-Precipitation，DR-Distance to Road,PD-Population Density;* indicates bivariate enhancement, and ** indicates nonlinear enhancement).

	**CS vs WY**	**CS vs SC**	**CS vs HQ**	**CS vs FSV**	**WY vs SC**	**WY vs HQ**	**WYvsFSV**	**SC vs HQ**	**SC vs FSV**	**HQ vs FSV**
DEM∩Slope	0.065**	0.085**	0.175**	0.086**	0.249**	0.223**	0.090**	0.202**	0.258**	0.225**
DEM∩DR	0.116**	0.119**	0.175**	0.125**	0.275**	0.322**	0.102**	0.284**	0.254**	0.328**
DEM∩PD	0.109**	0.097**	0.139**	0.115**	0.227*	0.192**	0.092**	0.185**	0.241**	0.214**
DEM∩P	0.071**	0.085**	0.141**	0.086**	0.322*	0.189*	0.090**	0.171**	0.241*	0.198*
DEM∩DHS	0.109**	0.127**	0.278**	0.138**	0.342**	0.213**	0.108**	0.233**	0.242**	0.259**
DEM∩FT	0.910*	0.937**	0.413**	0.934**	0.645**	0.351**	0.352**	0.544*	0.236*	0.563*
Slope∩DR	0.112**	0.102**	0.123**	0.111**	0.116**	0.191**	0.127**	0.165**	0.119**	0.165**
Slope∩PD	0.101**	0.079**	0.059**	0.104**	0.115*	0.065**	0.062**	0.064**	0.067**	0.052**
Slope∩P	0.109**	0.114**	0.048**	0.111**	0.227*	0.128**	0.087**	0.089**	0.146**	0.096**
Slope∩DHS	0.093**	0.077**	0.152**	0.079**	0.212**	0.113**	0.081**	0.110**	0.066**	0.093**
Slope∩FT	0.898*	0.935**	0.236**	0.928**	0.619**	0.267**	0.297**	0.499**	0.184**	0.466**
DR∩PD	0.086*	0.006*	0.035**	0.088*	0.153**	0.138**	0.071**	0.099**	0.082**	0.093**
DR∩P	0.108**	0.096**	0.069**	0.113**	0.259**	0.157**	0.064**	0.096**	0.136**	0.104**
DR∩DHS	0.142**	0.119**	0.230**	0.123**	0.259**	0.146**	0.092**	0.148**	0.101**	0.151**
DR∩FT	0.906*	0.933**	0.228**	0.932**	0.616**	0.308**	0.321**	0.498**	0.091**	0.475*
PD∩P	0.063**	0.045**	0.028**	0.679*	0.222*	0.059*	0.074**	0.012**	0.121**	0.017**
PD∩DHS	0.092**	0.058**	0.138**	0.086**	0.248**	0.072**	0.097**	0.079**	0.092**	0.067**
PD∩FT	0.897*	0.932**	0.201**	0.928**	0.619**	0.231**	0.283**	0.462**	0.091**	0.441**
P∩DHS	0.079**	0.093**	0.168**	0.074**	0.285*	0.135**	0.047**	0.121**	0.131**	0.131**
P∩FT	0.905*	0.935**	0.198**	0.928**	0.739**	0.269**	0.347**	0.462**	0.173**	0.449**
DHS∩FT	0.913*	0.935**	0.268**	0.930**	0.718**	0.255**	0.426**	0.510**	0.122**	0.499**
